# MnSOD and GPx1 polymorphism relationship with coronary heart disease risk and severity

**DOI:** 10.1186/s40659-016-0083-6

**Published:** 2016-04-11

**Authors:** Yosra Souiden, Hela Mallouli, Salah Meskhi, Yassine Chaabouni, Ahmed Rebai, Foued Chéour, Kacem Mahdouani

**Affiliations:** Laboratory of Biochemistry and Molecular Biology, Hospital of Ibn Eljazzar of Kairouan, Avenue Ibn Eljazzar, 3140 Kairouan, Tunisia; Laboratory of Analysis, Treatment and Valorization of the pollutants of the environment and products, Faculty of Pharmacy, Rue Ibn Sina, 5000 Monastir, Tunisia; Department of Cardiovascular, Hospital of Ibn Eljazzar of Kairouan, Avenue Ibn Eljazzar, 3140 Kairouan, Tunisia; Laboratory of Molecular and Cellular Screening Processes, Center of Biotechnology of Sfax, P. O. Box 1177, 3018 Sfax, Tunisia; Institute of Applied Biology of Medenine, 4119 Medenine, Tunisia

**Keywords:** Genetic polymorphism, Coronary heart disease, SOD activity, GPx activity, Total antioxidant status, Atherosclerosis

## Abstract

**Background:**

Disturbance of the equilibrium between reactive oxygen species (ROS) and anti-oxidants (AOX) has been implicated in various diseases, including atherosclerosis, the most common pathologic process underlying coronary heart disease (CHD). Thus, the defense systems against ROS are critical protecting blood vessel walls against oxidative damage. In this study, we investigate whether Ala16Val MnSOD and Pro198Leu GPx polymorphisms are associated with CHD susceptibility and/or severity.

**Methods:**

Both polymorphisms were genotyped in a sample of 203 controls and 164 patients. CHD risk and severity, antioxidant status (enzymatic and/or non enzymatic) and biochemical parameters were assessed and analysed by genotype.

**Results:**

A significant association of MnSOD variant to CHD risk was revealed in males. Males harboring the Val/Val genotype were approximately at twofold increased risk of CHD compared to controls (Ala carriers vs Val/Val, adjusted OR 1.89; 95 % CI 1.18‒3.42, p = 0.03). Significant decreases in SOD activity and total antioxidant status (TAS) were observed in Val carriers and by CHD status. Whereas, no association of GPx variant genotype (Leu/Leu) and activity to cardiopathy events was discerned. CHD severity, as demonstrated by the number of vessel stenosis, was associated with significantly higher frequency of Val allele and LDL levels in CHD subjects.

**Conclusions:**

Our results showed a lack of association of Pro198Leu GPx polymorphism to CHD risk and severity. However, they suggest that Ala16Val MnSOD polymorphism and decreased antioxidant defences are likely contributed to CHD risk in Tunisian men. Furthermore, the Val encoding MnSOD allele and decreased SOD activity were significantly correlated with CHD stenosis progression.

## Background

Coronary Artery Disease is the major cause of mortality and morbidity worldwide [1]. It is independently associated with various risk factors such as advanced age, hypertension, smoking habit, diabetes mellitus, hyperlipidemia, positive family story, obesity and inactivity [2].

Atherosclerosis, the most common pathologic process underlying coronary heart disease, represents a state of heightened oxidative stress characterized by endothelial dysfunction and plaque disruption. Oxidative stress can occur when the balance is upset, either by an excessive production of reactive oxygen species (ROS), by deficient antioxidant defenses, or by the combination of both [3]. In such circumstances, ROS may interact with cellular bio-molecules, leading to modification and potentially serious consequences for the cell [4].

Several potentially significant genetic variants related to oxidative stress have been already identified [5, 6]. In this study, we are interested to polymorphisms of the superoxide dismutase (SOD) and glutathione peroxidase (GPx) in order to check their possible implication in CHD risk and severity.

SOD is the primary antioxidant in the mitochondria that converts ROS into oxygen and hydrogen peroxide [7–10]. There are three SOD isoforms, including the mitochondrial SOD manganese dependent (MnSOD). MnSOD is encoded by a single gene containing five exons and it is located on chromosome 6q25 [11]. One of the common polymorphisms of MnSOD results in the replacement of alanine 16 (*GCT*) with a valine (*GTT*); the *Ala16Val* polymorphism. This polymorphism affects the import of MnSOD into the mitochondria by altering the conformation of its leader signal [12]. This mutation may reflect a functional polymorphism of mitochondrial transport of human MnSOD. It has been shown that the *16Ala* variant allows efficient targeting of MnSOD to the mitochondria, as evidenced by Sutton et al. [13] who found that it was 30–40 % more efficiently localized to the mitochondria than the 16Val variant. This is related to the fact that 16Ala variant has an α-helix structure that is easily imported and it reaches high levels of mitochondrial concentration and activity, whereas the 16Val variant has a partial ß-sheet structure that is partly stuck within the narrow inner membrane import pore and is subsequently degraded by the proteasome. Furthermore, the mRNA that encodes the 16Val variant is more rapidly degraded than the Ala variant [14].

The second line of enzymatic antioxidant defense is played by glutathione peroxidase (GPx) isoenzymes. GPx has 6 isoforms (GPx-1-6) [15]. GPx-1 is the most widely distributed and abundant form in human cells, including vascular endothelium [16]. GPx-1 is an intracellular soluble selenoprotein which converts peroxides such as H2O2 and ROOH into water and alcohol [17]. The gene coding for GPx-1, is located on chromosome 3p21.3 [18] and it is composed of 2 exons with a 1.42 kb region [19]. Several polymorphisms have been described in the GPx-1 gene. One of them is located at codon 198 (C > T) resulting in an amino acid variation from proline (CCC) to leucine (CTC). This amino acid substitution causes a change of the structural conformation of the active site region and modifies the enzyme activity [20]. Previous studies using aortic endothelial cells demonstrated a 40 % reduction in the GPx-1 activity being associated with the T allele and showed that the Pro198Leu variant was associated with increased carotid intima-to-media thickness, peripheral arterial disease, and increased CAD risk [21–24].

The aim of our study was to assess the association of MnSOD and GPx polymorphisms and activities with CHD risk and severity in the Tunisian population.

## Methods

### Study population

One hundred and sixty-four patients with coronary heart disease (CHD) and two hundred and three controls were enrolled in this study from June to September 2011. The healthy subjects were recruited at their annual health examination at Hospital Ibn Eljazzar, Kairouan, Tunisia, and did not have any chest symptoms or electrocardiogram (ECG) abnormalities suggesting CHD, or a medical history for CHD.

Patients were admitted to the cardiovascular department of Hospital Ibn Eljazzar, Kairouan, Tunisia. The diagnosis of Acute CHD was based on the presence of at least 2 of the following three elements: (1) Ischemic type of chest pain (2) changes on serial electrocardiogram (ECG) tracings (3) Increase in serum cardiac marker [elevated creatine kinase isoenzyme MB (CK-MB) and troponin T within 12 h after the onset of pain]. Patients with associated renal failure, liver disease, lung disease, pregnancy, thyroid disease, gastrointestinal disease were excluded. The study protocol was approved by the local Ethics Committee of the hospital Ibn Eljazzar. Written informed consent was obtained from all subjects before their participation in the study.

All participants were interviewed, and data on hypertension, diabetes mellitus, dyslipidemia, medical history including family history, smoking status and duration of CHD were recorded. For coronary risk factors the following definitions were used: individuals were defined as hypertensive if their blood pressure was >140/90 mmHg or in case of self-reported use of an antihypertensive drug. Diabetes mellitus was diagnosed if the fasting plasma glucose concentration was ≥7 mmol/l or if the patient was treated with insulin or oral hypoglycemic agents; individuals were deemed dyslipidemic when their total cholesterol concentration was ≥5.68 mmol/l, or their triglyceride concentration was ≥2.28 mmol/l, or they receive lipid-lowering drugs. Smoking was coded as never and current smoker. Gender and body mass index (BMI) were also recorded.

### Blood collection

From all subjects, Blood samples were collected in heparinized (5 ml) and EDTA (5 ml) tubes after 12 h fasting and immediately centrifuged at 1600×*g*. After separation, washed and lysed erythrocytes as well as plasma were stored at −70 °C until biochemical measurements were performed.

### Biochemical analyses

#### Superoxide Dismutase (SOD) Assay

The proportioning of erythrocyte enzymatic activity superoxide dismutase (SOD) is based on velocity measurement of oxidation reaction inhibition of I.N.T. (2-(4-Iodophenyl)-3-(4-nitrophenol)-5-phenyltetrazoliumn chloride) by SOD. The role of SOD is to accelerate the dismutation of toxic superoxide radical (O2−) produced during oxidative energy processes to hydrogen peroxide and molecular oxygen. This method uses xanthine and xanthine oxidase (XOD) to generate superoxide radicals which react with I.N.T. to form a red formazan dye. SOD activity is then measured by the degree of inhibition of this reaction. The results were expressed as U/g Hb.

#### Glutathione peroxidase (GPx) assay

The erythrocyte hemolysate GPx1 activity was assessed by Paglia and Valentine method [25] using the Ransel kit (Randox, Antrim, UK) based on the oxidation reduction of glutathione and the measurement of the variations of reduced NADP. GPx catalysis the oxidation of glutathione (GSH) by cumene hydroperoxide. In the presence of glutathione reductase and NADPH oxidized glutathione (GSSG) is immediately converted to reduced form with a concomitant oxidation of NADPH to NADP+. The enzyme activity is measured at a wavelength of 340 nm. One unit of GPX activity is defined as the amount of the enzyme required for oxidizing 1 μmol of NADPH per minute. Results were expressed in units per gram of hemoglobin.

#### Total antioxidant status (TAS) assay

Total antioxidant status (TAS) is measured by the reduction in color produced by a radical cation (ABTS^®+^). The assay principle is as follow: the ABTS^®^ (2,2′-azino-di-[3-ethylbenzthiazoline sulphonate]) is incubated with metmyoglobin which has peroxidase activity and H_2_O_2_ to produce the radical cation ABTS^®+^. This has a relatively stable blue-green color measured at 600 nm. Antioxidants in the added sample cause suppression of this color production to a degree which is proportional to their concentration.

#### Other analyses

Measurements of other biochemical parameters tested during this study included heparinized plasma triglycerides, cholesterol, HDL-Cholesterol, were detected by an automate (Konelab 20, Thermo Clinical Labsystems Oy, Finland). LDL-Cholesterol was calculated using the Friedewald equation.

### Genetic analyses

**Fig. 1:**
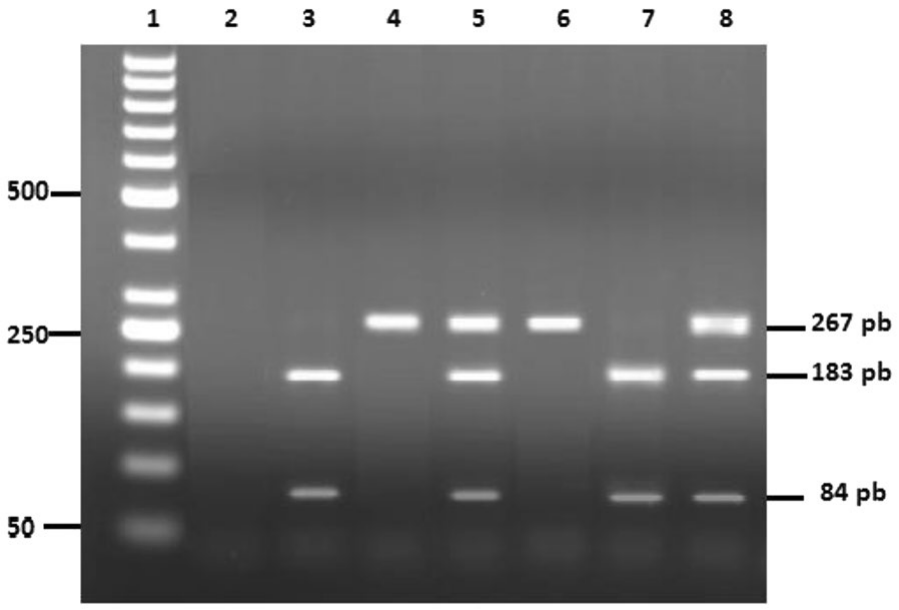
Restriction fragments of MnSOD gene amplified products. *Lane1* DNA molecular weight marker (50 pb); *lane 2* negative control; *lanes 3* and *7* Val/Val mutated homozygous genotype; *lanes 4* and *6* Ala/Ala wildtype genotype; *lanes 5* and *8*, Ala/Val heterozygous genotype

Genomic DNA was isolated from 300 µl of peripheral blood leukocytes using Promega DNA isolation kit according to the manufacturer’s recommendations. An alanine/valine polymorphism in the signal peptide of *Mn*-*SOD* gene was evaluated by PCR–RFLP analysis according to the method described by Ibrahim et al. [26]. PCR amplifications were performed in a total volume of 20 μl containing 50 ng of genomic DNA, 1.25 U Taq polymerase, 2 mM dNTP, 2 mM MgCl_2_ and 1X PCR buffer in the presence of 0.4 μmol/l of each primer (forward 5′-CAG CCC AGC CTG CGT AGA CGG-3′ and reverse 5′-CTT GGC CAA CGC CTC CTG GTA CTT-3′) to amplify a 267-bp fragment. The PCR conditions involved an initial denaturation of DNA at 95 °C for 5 min, followed by 30 cycles of amplification at 95 °C for 45 s (melting), 54 °C for 30 s (annealing) and 72 °C for 30 s, and a final extension at 72 °C for 5 min. The resulting 267-bp PCR product was digested with the restriction endonuclease BsaW1 at 37 °C for 2 h according to the manufacturer’s recommendations and digested products were visualized with electrophoresis in 2.5 % agarose gel stained with ethidium bromide (0.5 µg/ml). Restriction enzyme digestion results in a 267-bp product (16Ala) or 183 and 84 bp products (16Val) (Fig. 1).

**Fig. 2:**
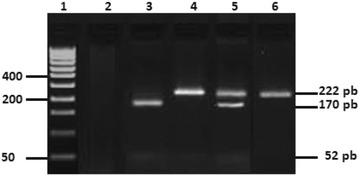
Restriction fragments of GPx1 gene amplified products. *Lane 1* DNA molecular weight marker (50 pb); *lane 2* negative control; *lane 3* Pro/Pro wildtype genotype; *lanes 4* and *6* Leu/Leu mutated homozygous genotype; *lane 5* Pro/Leu mutated heterozygous genotype

The GPx1 198Pro/Leu variant was determined using 5′-TCC AGA CCA TTG ACA TCG AG-3′ (forward) and 5′-ACT GGG ATC AAC AGG ACC AG-3′ (reverse) primers. A 222 base pair DNA fragment containing the polymorphic site was amplified in a total volume of 20 μl, containing 2 μl 10xPCR buffer, 1.125 mmol/l MgCl2, 0.15 mmol/l dNTPs, 0.25 μmol/l each primer, 100 ng of genomic DNA and 1.5 U of Taq DNA polymerase (Promega Madison, USA). The PCR amplification was performed in 35 cycles at 94 °C for 30 s, 59 °C for 30 s and 72 °C for 30 s, preceded by an initial denaturation at 94 °C for 8 min and followed by a final elongation step at 72 °C for 7 min. The amplicon was then digested with ApaI (Promega Madison, USA) restriction enzyme and resolved in 2 % agarose gel. The digested products showed 2 fragments of 170 and 52 bp for the 198Pro wildtype homozygous, 3 fragments of 222, 170 and 52 for the 198Pro/Leu (CT) heterozygote, and 1 fragment of 222 pb for the 198Leu (T) mutated homozygous genotype (Fig. 2).

### Statistical tests

Statistical analyses were performed using the Statistical Package for the Social Sciences (SPSS) version 17.0 for Windows (SPSS, Chicago, IL), R software version 3.0.2 and QUANTO program version 1.2. The Chi square test was used to test the distribution of genotypes for deviations from Hardy–Weinberg equilibrium. genotype distribution of *MnSOD* and *GPx1* gene in all subjects was analyzed by Chi square test. Distributions of continuous variables in groups were expressed as mean ± SD.

Comparison between two groups was performed using two-tailed *t* tests. Comparison between more than 2 groups was performed by one-way analysis of variance (ANOVA). Simple associations between variables were calculated as the Pearson correlation.

Logistic regression was used to evaluate the effect of MnSOD genotypes and activity, after adjusting for other potential confounders such as age, gender, BMI and smoking status. p < 0.05 was required for statistical significance.

Empirical power to detect the association provided by our sample was computed using an R script with 10,000 permutations. Theoretical power was also calculated using Quanto and parameter values (Odds ratio, allele frequencies and sample size) equals to those found in our study.

## Results

Three hundred and sixty-seven subjects were investigated in this study in order to evaluate the association of the MnSOD Ala16Val and GPx1 Pro198Leu polymorphisms with the coronary heart disease (CHD).

Characteristics of the enrolled subjects stratified according to MnSOD and GPx1 polymorphisms are illustrated in Table 1. Results observation showed a significant difference between MnSOD genotype classes associated with male gender and diabetes mellitus. Whereas, a significant difference between GPx1 genotype groups was noted only according to LDL level in the study population.

**Table 1:** Basic characteristics of the study population by genotype

Trait	SOD genotype				GPx genotype			
	Ala/Ala (CC) (N = 92)	Ala/Val (CT) (N = 172)	Val/Val (TT) (N = 103)	P	Pro/Pro (CC) (N = 180)	Pro/Leu (CC) (N = 152)	Leu/Leu (TT) (N = 35)	P
Age (years)	60.47 ± 10.88	62.42 ± 11.98	61.51 ± 11.99	0.43	61.57 ± 11.59	62.42 ± 12.24	63.37 ± 10.10	0.64
Body mass index (kg/m^2^)	25.90 ± 4.08	26.26 ± 3.72	26.42 ± 3.49	0.61	26.02 ± 3.63	26.45 ± 3.77	26.17 ± 4.25	0.58
Male, n (%)	61 (66.30 %)	88 (51.16 %)	67 (65.05 %)	*0.02*	107 (59.44 %)	91 (59.87 %)	18 (51.43 %)	0.64
Hypertension, n (%)	22 (23.91 %)	43 (25.0 %)	31 (30.10 %)	0.55	50 (27.78 %)	37 (24.34 %)	9 (25.72 %)	0.77
Hyperlipidemia, n (%)	16 (17.39 %)	27 (15.70 %)	29 (28.15 %)	*0.03*	26 (14.45 %)	22 (14.47 %)	10 (28.75 %)	0.09
Diabetes mellitus, n (%)	14 (15.22 %)	20 (11.62 %)	25 (24.27 %)	*0.02*	30 (16.67 %)	23 (15.13 %)	6 (17.14 %)	0.92
Current Smokers, n (%)	47 (51.08 %)	86 (50.0 %)	49 (47.57 %)	0.88	86 (47.78 %)	78 (51.32 %)	18 (51.43 %)	0.79
Plasma glucose (mmol/l)	5.71 ± 3.68	5.25 ± 2.26	5.62 ± 2.15	0.33	5.70 ± 3.27	5.24 ± 2.51	5.22 ± 2.68	0.32
LDL cholesterol (mmol/l)	2.52 ± 1.09	2.46 ± 1.02	2.48 ± 1.08	0.91	2.68 ± 1.03	2.42 ± 0.89	2.73 ± 1.13	*0.04*
Triglycerides (mmol/l)	1.57 ± 0.66	1.63 ± 0.70	1.66 ± 0.63	0.64	1.65 ± 0.58	1.64 ± 0.74	1.67 ± 0.66	0.97
HDL cholesterol (mmol/l)	1.32 ± 0.72	1.45 ± 0.74	1.30 ± 0.69	0.18	1.30 ± 0.75	1.40 ± 0.71	1.32 ± 0.72	0.52
CRP (mg/l)	1.50 ± 0.34	1.61 ± 0.44	1.58 ± 0.46	0.13	1.80 ± 0.53	1.73 ± 0.38	1.87 ± 0.66	0.22
Non enzymatic antioxidant parameters								
Uric Acid (µmol/l)	323.13 ± 120.6	313.69 ± 108.9	314.83 ± 132.6	0.82	319.4 ± 123.7	329.5 ± 123.5	323.1 ± 120.5	0.76
Total bilirubin (µmol/l)	14.12 ± 6.57	13.32 ± 6.52	13.16 ± 7.20	0.56	12.88 ± 6.90	13.50 ± 6.70	13.07 ± 6.32	0.71
Direct bilirubin (µmol/l)	5.34 ± 2.39	5.91 ± 2.09	5.53 ± 2.25	0.11	5.16 ± 2.14	5.37 ± 2.02	4.77 ± 2.71	0.28
Albumin (g/l)	42.46 ± 6.28	43.06 ± 4.47	41.88 ± 7.33	0.3	40.89 ± 5.99	41.44 ± 4.65	43.15 ± 10.47	0.14
Iron (µmol/l)	18.35 ± 5.07	17.34 ± 5.37	18.34 ± 6.32	0.23	18.58 ± 5.65	17.33 ± 4.91	18.07 ± 7.10	0.12
Enzymatic antioxidant parameters								
SOD activity (U/gHb)	1392.6 ± 219.3	1285.3 ± 188.9	979.4 ± 220.4	*<10* ^*−4*^	–	–	‒	‒
GPx activity (U/gHb)	–	–	–	–	40.22 ± 12.79	42.91 ± 13.17	39.01 ± 11.97	0.1
TAS (mmol/l)	1.51 ± 0.27	1.53 ± 0.31	1.42 ± 0.33	*0.006*	1.54 ± 0.29	1.50 ± 0.28	1.41 ± 0.35	*0.002*

*P < 0.05 was required for statistical significance

Biochemical examination of our population was carried out by testing several parameters (Table 1). Results showed a significant difference between genotype groups for antioxidant status. Indeed, SOD2 but not GPx1 activity decreased significantly in the presence of the mutated allele (Val and Leu, respectively). The same is true for total antioxidant status (TAS). In fact, significant reduced levels of TAS were detected in variant carrier groups. For other parameters, no differences were noted by genotype.

In the whole population, genotype distribution of the Mn-SOD Ala16Val (CC/CT/TT: 92/172/103, χ^2^ = 1.40, p = 0.24) and GPx1 Pro198Leu (CC/CT/TT: 180/152/35, χ^2^ = 0.13, p = 0.72) variants satisfied the Hardy–Weinberg equilibrium (p > 0.05) with a C allele frequency of 48.50 % and 69.75 % respectively for *MnSOD* and *GPx1* genes (Table 1).

Relationship between gene polymorphisms and CHD, as well as CHD risk estimation, were assessed by comparing Mn-SOD Ala16Val and GPx1 Pro198Leu genotype distribution among cases and controls (Tables 2, 3).

**Table 2:** Basic characteristics of the control and case groups by gender

Trait	Males (N = 216)			Females (N = 151)		
	Controls (N = 119)	Cases (N = 97)	P	Controls (N = 84)	Cases (N = 67)	P
Age (years)	58.68 ± 12.01	64.15 ± 10.71	<*10* ^*−4*^	63.38 ± 12.16	61.28 ± 11.00	0.27
Body mass index (kg/m^2^)	24.80 ± 3.41	25.23 ± 2.79	0.32	25.64 ± 3.68	24.93 ± 3.66	0.24
Hypertension, n (%)	0 (0.0 %)	65 (67.01 %)	<*10* ^*−4*^	13 (15.48 %)	18 (26.87 %)	0.09
Diabetes mellitus, n (%)	0 (0.0 %)	39 (58.21 %)	<*10* ^*−4*^	10 (11.90 %)	20 (29.85 %)	0.006
Plasma glucose (mmol/l)	4.56 ± 0.71	5.02 ± 3.09	0.12	4.37 ± 0.70	4.71 ± 2.13	0.17
LDL cholesterol (mmol/l)	2.55 ± 1.24	3.13 ± 1.14	*5E−04*	2.54 ± 1.14	3.49 ± 1.49	<*10* ^*−4*^
Triglycerides (mmol/l)	1.24 ± 0.73	1.21 ± 0.65	0.75	1.33 ± 0.47	1.47 ± 0.92	0.23
HDL cholesterol (mmol/l)	0.83 ± 0.22	0.84 ± 0.27	0.76	0.82 ± 0.44	0.79 ± 0.26	0.62
Non enzymatic antioxidant parameters						
Uric Acid (µmol/l)	332.3 ± 92.90	342.38 ± 154.27	0.55	285.21 ± 83.71	326.27 ± 138.95	*0.03*
Total bilirubin (µmol/l)	18.39 ± 4.21	8.11 ± 4.58	<*10* ^*−4*^	15.89 ± 5.49	7.62 ± 5.04	<*10* ^*−4*^
Direct bilirubin (µmol/l)	7.93 ± 3.14	2.71 ± 1.95	<*10* ^*−4*^	6.83 ± 3.72	2.07 ± 1.74	<*10* ^*−4*^
Albumin (g/l)	41.72 ± 4.23	40.65 ± 4.30	0.07	41.75 ± 7.83	40.95 ± 4.10	0.45
Iron (µmol/l)	18.47 ± 4.54	16.20 ± 3.90	*10* ^*−4*^	17.93 ± 5.41	16.60 ± 3.10	0.075
Enzymatic antioxidant parameters						
SOD activity, (U/gHb)	1321.4 ± 204.8	1089.2 ± 219.7	<*10* ^*−4*^	1463.8 ± 233.4	1166.5 ± 213.9	<*10* ^*−4*^
TAS (mmol/l)	1.63 ± 0.271	1.40 ± 0.232	<*10* ^*−4*^	1.70 ± 0.267	1.38 ± 0.289	<*10* ^*−4*^
SOD genotypes (%), n (CC/CT/TT)	31.93/45.38/22.69 %	23.71/37.11/39.18 %	*0.03*	17.86/55.95/26.19 %	23.88/52.24/23.88 %	0.66
	38/54/27	23/36/38		15/47/22	16/35/16	

*P < 0.05 was required for statistical significance

**Table 3:** GPx polymorphism and parameter stratification by CHD status

Trait	Controls (203)	Cases (164)	P
Age (years)	60.63 ± 12.27	62.98 ± 11.89	0.07
Body mass index (kg/m^2^)	24.73 ± 3.52	28.05 ± 3.17	<*10* ^*−4*^
Male, n (%)	119 (58.62 %)	97 (59.15 %)	0.92
Hypertension, n (%)	13 (6.40 %)	96 (58.54 %)	<*10* ^*−4*^
Diabetes mellitus, n (%)	10 (4.93 %)	59 (35.98 %)	<*10* ^*−4*^
Plasma glucose (mmol/l)	4.33 ± 0.71	4.47 ± 2.91	0.51
LDL cholesterol (mmol/l)	2.54 ± 1.12	3.28 ± 1.13	<*10* ^*−4*^
HDL cholesterol (mmol/l)	0.83 ± 0.37	0.82 ± 0.27	0.77
Triglycerides (mmol/l)	1.24 ± 0.64	1.31 ± 0.77	0.34
Non enzymatic antioxidant parameters			
Uric Acid (µmol/l)	312.96 ± 92.00	335.82 ± 147.99	0.07
Total bilirubin (µmol/l)	17.04 ± 4.73	7.68 ± 4.81	<*10* ^*−4*^
Direct bilirubin (µmol/l)	7.34 ± 3.21	2.36 ± 1.08	<*10* ^*−4*^
Albumin (g/l)	41.74 ± 6.05	40.78 ± 4.18	0.09
Iron (µmol/l)	18.23 ± 4.94	16.38 ± 3.47	<*10* ^*−4*^
Enzymatic antioxidant parameters			
GPx activity, (U/gHb)	42.17 ± 13.99	40.20 ± 12.25	0.16
TAS (mmol/l)	1.68 ± 0.26	1.37 ± 0.25	<*10* ^*−4*^
GPx genotypes (%), n (CC/CT/TT)	43.84/44.34/11.82 %	55.49/37.80/6.71 %	0.051
	89/90/24	91/62/11	0.1
GPx genotypes (%), n (CC + CT/TT)	88.18/11.82 %	93.29/6.71	
	179/24	153/11	

*P < 0.05 was required for statistical significance

Taking into account the significant difference detected between Mn-SOD genotype groups associated to some risk factors (Table 1), genotype distribution in control and case groups was studied by gender (Table 2). Several compounds proposed to act as antioxidants (AOX) in vivo [27] were considered in our study. We found a significant decrease in direct and total bilirubin in both male and female cases. Moreover, a significant difference at iron level was observed between controls and cases in the male group. However, in female group, an increase of uric acid was noted. No significant difference at albumin level was observed between cases and controls of male and female groups. SOD activity and TAS were measured in both control and CHD groups. In harmony with the first results obtained in the whole population after genotype stratification (Table 1), significant differences were detected in male and female groups between cases and controls. In line with these results, comparison of MnSOD genotype distribution by CHD status and gender, enables us to detect a significant difference between controls and cases of male group (controls vs cases: 31.93/45.38/22.69 vs 23.71/37.11/39.18 %, p = 0.03). The statistical analysis showed an association between the MnSOD polymorphism and the risk of CHD among men (Ala carriers vs Val/Val, OR 2.19, 95 % CI 1.21‒3.97, p = 0.009).

Since CHD is a multifactor pathology, the association of Val variant to the risk of CHD may be influenced by a number of risk factors. Accordingly, the significance of the mutation can be overestimated. As shown in Table 2, compared with controls, men with CHD had increased age, higher prevalence of diabetes and hypertension, and elevated LDL level. Adjustment for the last possible confounders by a multivariate regression analysis demonstrated that the association persisted (Ala carriers vs Val/Val, adjusted OR 1.89; 95 % CI 1.18‒3.42, p = 0.03).

Association between GPx polymorphism and CHD risk was examined. Baseline characteristic stratification according to GPx genotype showed no differences in clinical, anthropometric and biochemical parameters except for LDL. An elevation of LDL level was noted among the TT genotype group. Moreover, significant decreases in TAS levels but not in GPx activities was discerned among T carriers (Table 1). Based on these findings, we explored the association between T allele, lipid and antioxidant parameters further after stratifying by CHD status (Table 3). On one hand, results observation revealed a significant increase of plasma LDL level among T carriers in the cases group compared to the control one, on the other, a significant decrease in total and direct bilirubin, iron and TAS level but not in GPx activity was noted in the same group (p < 0.05). However, we observed no difference in genotype distribution between CHD and control groups (Controls vs CHD 55.49/37.80/6.71 %; 43.84/44.34/11.82 %, p = 0.051). Statistical analysis showed a lack of association of GPx1 Pro198Leu polymorphism with CHD risk (Pro carriers vs Leu/Leu, OR 0.54, 95 % CI 0.25‒1.13, p = 0.10).

The assessment of the power provided by our sample size to detect allele association was performed for each variant. Given a sample size of 164 control subjects and 203 patients with CHD, our study had an empirical power of 30 and 43 % respectively for MnSOD Ala16Val and GPx1 Pro198Leu polymorphisms. Whereas, the current study had a theoretical power of 97 % to detect an OR of 1.80 associated with each variant, assuming an additive effect of alleles and with a 5 % type 1 error rate. In this sample, a size effect of approximately 1.52 is detectable with 80 % power.

In the light of our first findings of high LDL levels among case group, the relationship between MnSOD/GPx1 activities, genotype and lipid parameters was studied in a group of 106 subjects who underwent coronary angiography (Table 4).

**Table 4:** Lipid parameters, enzymatic antioxydant activity/polymorphism and CHD severity

Parameters	0-vessel (n = 13)	1-vessel (n = 42)	2-vessels (n = 35)	3-vessels (n = 16)
Total cholesterol (mmol/L)	4.11 ± 1.03	4.26 ± 1.07	4.59 ± 1.03	5.36 ± 1.41*
HDL-cholesterol (mmol/L)	0.86 ± 0.28	0.84 ± 0.26	0.81 ± 0.29	0.79 ± 0.23
LDL-cholesterol (mmol/L)	3.02 ± 0.93	3.15 ± 1.01	3.49 ± 1.04	4.19 ± 1.39*
Triglycerides (mmol/L)	1.14 ± 0.63	1.27 ± 0.65	1.34 ± 0.97	1.89 ± 1.00*
SOD activity, (U/gHb)	1246.9 ± 242.15	1112.4 ± 141.37	1065.6 ± 194.28	864.3 ± 229.08**^, ‡^
SOD genotypes, n (%), (CC/CT/TT)	4/6/3	10/21/11	7/18/10	3/7/6
	(30.77/46.15/23.08)	(23.81/50.0/26.19)	(20.0/51.43/28.57)	(18.75/43.75/37.5)
GPx activity, (U/gHb)	38.90 ± 7.89	44.09 ± 11.32	42.94 ± 11.15	43.65 ± 12.64
GPx genotypes, n (%), (CC/CT/TT)	7/8/1	22/17/3	21/12/2	9/6/1
	(53.85/38.46/7.69)	(52.38/40.47/7.14)	(60.0/34.29/5.71)	(56.26/37.5/6.25)

* P < 0.05 compared with 0-vessel disease and P = 0.001 with 1-vessel disease

** P < 0.001 compared with 0-vessel disease and with 1-vessel disease and P = 0.002 with 2-vessels disease

^‡^ P < 0.001 compared with 0-vessel disease, 1-vessel disease and 2-vessels disease

CHD group was classified into four subgroups according to the number of affected coronary arteries (Table 4). Plasma levels of lipid parameters tended to be increased with more severe coronary atherosclerosis. Lower SOD activity was significantly associated with the severity of CHD (p < 0.05). We noted graded reduced SOD activities in patients presenting 1–3 vessel stenosis. However, no differences in GPx activities were observed between stenosis subgroups (p = 0.53).

Pearson correlation study between MnSOD activity and lipid parameters showed a positive correlation with high density lipoprotein (HDL) (r = 0.143, p = 0.028), and a negative correlation with low density lipoprotein (LDL) (r = −0.210, p = 0.034). Nonetheless, no correlation between the two parameters and GPx activity was observed.

Exploration of the relationship between enzyme activities and genotypes through CHD pathogenesis showed, in contrast to GPx activity, an association of decreased SOD activity to the frequent presence of variant genotype (Val/Val) as increased stenosed vessel number.

Multivariate regression analysis stratified by gender and adjusted according to age, body mass index hyperlipidimia and smoking habits as covariates, confirmed the dichotomous age (p = 0.012), hyperlipidemia (p = 0.027) effect of the Mn-SOD Ala16Val polymorphism on CHD severity.

## Discussion

A growing body of evidence supports the concept that oxidative stress plays critical roles in the initiation and progression of numerous diseases including atherosclerosis [28]. Since the vulnerability to oxidative stress is partly determined by genetic background, there have been several studies examining the association between functional gene polymorphisms of the key enzymes of redox regulation and the risk of CAD.

To our knowledge, this is the first study to investigate the relationship between Mn-SOD/GPx1 polymorphisms, and CHD risk and severity in a Tunisian population. MnSOD and GPx1 genotype distributions in control and patient groups were examined. The comparison of genotypic frequencies in our control group (TT genotype: 26.11 and 9.54 %, respectively) with those reported in other studies showed that our results agree with those of the Caucasian population (26.11 and 9.54 %, respectively) [29–31] but differ from those reported in Asian (66–79.4 and 7–13 %, respectively), Afro-Caribbean (42.9 and 18 %, respectively) and oriental (77.8 and 0.0 %, respectively) population [21, 31–39]. This clear difference, mostly related to T allele, is evident, suggesting that this allele may have a differential role in the disease process in these ethnically distinct populations. In fact, in the studied control group, T allele frequencies of MnSOD and GPx1 genes were equal to those mentioned in previous studies of Caucasian population (49 % [48–51 %] and 34 % [31–36 %], respectively), but unlike those of Asian population (13 % [5–21 %] and 23 % [18–29 %], respectively) [20, 31, 40–42].

Given their role in antioxidant defense, SOD2 and GPx1 genes are considered as attractive low penetrance candidate genes for CHD. In this study, we examined the impact of SOD2 Ala16Val and GPx1 Pro198Leu polymorphisms in CHD risk and severity. Our results indicated that the Val/Val genotype of MnSOD envisages in Tunisian men higher risk to CHD as compared to controls (Table 2). In fact, Val/Val genotype was significantly more common among case (39.18 %) than control (22.69 %) men. Accordingly, we note that SOD2 polymorphism has a significant influence on CHD risk in Tunisian men (Ala carriers vs Val/Val, adjusted OR 1.89, 95 % CI 1.18‒3.42, p = 0.03). Furthermore, the observed difference between CHD and control men is unlikely to be the result of biased population sampling. There was no significant difference in our frequency estimation of the Val allele (45.38 %) from the Tunisian men of control group than those of the Caucasian population (44–48 %) reported in other studies [29, 30].

Our findings concur with those reported by two recent meta-analyzes in which Crawford et al. [28] concluded that the Val/Val or Val allele has been positively associated with cardiovascular disease risk and with comorbidities in type 1 (T1DM) and type 2 diabetes mellitus (T2DM) patients, and Tian et al. [43] showed a significant association of the C allele with reduced risk of CAD. Furthermore, in agreement with our data, Valenti et al. [44] reported an increased risk of cardiomyopathy in hemochromatosis patients with Val allele compared with Ala allele. Evenly, Fujimoto et al. [45] found that individuals harboring the valine variant were at increased risk for CAD and acute myocardial infraction. Moreover, in a large study of 776 Caucasian subjects with diabetes, Jones et al. [34] showed an increased risk of CHD associated to Val/Val genotype. Besides, in 24 of 100 patients recruited with severe heart failure, Charniot et al. [46] revealed a significant correlation of the Val-encoding MnSOD allele with the severity and prognosis of cadiogenic shock.

In contrast to SOD2 polymorphism, previous studies on the association between CHD and GPx1 polymorphism have been highly inconsistent. Polymorphic variation in GPx1 gene at position 198 is associated in some, but not in all studies. Our observations suggest that Pro198Leu GPx1 polymorphism was not associated to CHD risk. In line with our findings, previous Caucasian population studies revealed no significant association between allele frequency and risk to suffer from CAD. Indeed, in a French population, Charniot et al. [46] declared that GPx—variants influenced neither GPx activity nor cardiac events. Moreover, in a recent study of Iranian population, Najafi et al. [47] showed that GPx1 activity and rs1050450 (Pro198Leu) site are not involved in the development of coronary artery stenosis. However, in disagreement with our data, other studies performed on Asian population demonstrated an association of GPx1 variant genotypes to CHD risk and carotid artery intra-media thickness (IMT). In a Japanse population, Haminichi et al. [21] and Oguri et al. [39] suggested that functional variants in GPx1 gene are associated with increased carotid IMT of cardiovascular and peripheral vascular diseases in type 2 diabetic patients. Furthermore, data of a study carried out by Tang et al. [48], provide evidence that GPx1 Pro198Leu variant genotypes are significantly associated with CAD risk in a Chinese population. In fact, compared to the 198Pro/Pro carriers, subjects with the variant genotypes (198Pro/Leu and 198Leu/Leu) had a significantly higher risk of CAD (adjusted OR 2.02, 95 % CI 1.27–3.22). In the same way, a recent meta-analysis confirms the association of GPx1 variants with CAD in people with type 2 diabetes mellitus of Chinese and Indian populations [49].

The opposing findings of these reports may be attributable to inaccurate genotype frequency estimates resulting from small sample sizes, selection of control group, case characterization linkage disequilibrium between SOD2 and/or GPx1 alleles with different functionally relevant polymorphisms in specific ethnic groups and gene-environment interactions. Further variations in genetic background, dietary habits and/or environmental factors between different populations might account for these findings.

In this study, in addition to polymorphism association to CHD risk, we investigated the SOD and GPx activities in CHD subjects. Consistent with our results some studies suggested an association between SOD activity [34] but not GPx activity [31, 46, 47] with cardiopathy events. In contrast, others proposed the opposite [50–52]. We have observed a significant decrease in the SOD activity in patients with CHD, as compared to controls. We confirmed known fact that erythrocyte SOD activity is significantly reduced in patients with CHD [51, 53–55]. Lower enzyme activity could be a consequence of the increased oxidative stress induced by the coronary events. It may be the leading cause of post-translational covalent modifications in SOD e.g. nitration, phosphorylation, glutathionylation and glycation, which results in decreased enzyme activity [56].

Our results also showed no differences in GPx activity between genotype classes and according to CHD status. Indeed, normal GPx activity was measured in Leu carriers. These findings can be explained by the GPx upreglation under oxidative stress conditions and/or the presence of a compensatory mechanism in other cells of the body with faster turnover. In addition, through an analysis of the predicted tertiary structures, Jones et al. [34] provided another possible explanation. They showed that the polymorphic variants have no essential role on protein stability and function. They suggested that this may be due to their surface location.

Antioxidant status of the study population was further explored by measurement of some non-enzymatic antioxidants (AOX) and total antioxidant status (TAS). Significant decreases in both total and direct bilirubin as well as in iron against no differences in albumin and uric acid levels, were noted in cases compared to controls. Taken together, these non-enzymatic antioxidant (AOX) constitute an important aspect of a network essential for assessing in vivo AOX status [57]. In our population, their reduced values reflect a state of an imbalance between AOX and pro-oxidants and/or free radicals. The assessment of the TAS of a biological fluid is a composite measurement of the combined effects of individual scavenging AOX within the sample and providing insight into the overall prooxidant-antioxidant balance [58]. In our population, a global evaluation of the TAS showed a significantly reduced levels in CHD subjects compared to controls. Our result is in agreement with that of Nojiri et al. [59] but not with those of Alamdari et al. [60] and Rahsepar et al. [61] who found that the level of prooxidant/antioxidant balance (PAB) in patients with stable CAD was significantly higher compared to that measured in healthy control subjects.

Low TAS levels could reflect either high oxidative stress or decrease of defense against it. In CHD patients, even in stable cases, a high oxidative stress status has been reported [62–65]. For instance, circulating oxidized low density lipoprotein levels are positively associated with severity of acute coronary syndromes [66, 67] and with subclinical CHD [68]. In line with these findings, in our population, the CHD severity, as demonstrated by the number of vessel stenosis, was associated with high LDL levels. The observed positive correlation between atherosclerosis progression and high levels of LDL-cholesterol in CHD patients was associated with the SOD2-Val/Val genotype. This has not been confirmed by another study focused on the role of antioxidant enzymes in determining genetic susceptibility to the coronary artery disease in patients with T2DM [69]. Nevertheless, Kakko et al. [70] reported that carotid artery intima-media thickness, was greater in women with the Val allele and high levels of low-density lipoprotein (LDL) cholesterol (p = 0.03). Similarly, in patients with oxidised LDL <0.5 nmol/mg, Gottilieb et al. [71] revealed an association of Ala/Val and Val/Val genotypes with increased levels of oxidised LDL compared with Ala/Ala genotype in the same group. Furthermore, in a cohort of patient with cardiogenic shock due to dilated cardiomyopathy without acute coronary syndrome, the Val-encoding MnSOD allele was significantly correlated with the severity and prognosis of cardiogenic shock [46].

Predisposing association of the Val allele and high LDL-cholesterol levels in subjects with two or three stenosis vessels, showed that high LDL levels are more harmful in subjects with a diminished antioxidant capacity of MnSOD. Consistent with our data, a number of studies reported an association between antioxidant enzyme activities and progression of stenosis [45, 46, 59, 70, 72]. This could be explained on the one hand, by the decline resistance against ROS produced in the mitochondria due to the Val isoform of the SOD2, and on the other hand, it might be due to SOD activity inhibition by lipid peroxidation products [73, 74]. In this context, Botto et al. [75] have reported elevated levels of oxidative DNA damage in patients with angiographically documented CAD. In addition, it has been reported that isoprostanes, markers of lipid peroxidation, and reduced antioxidant capacity are related to increased risk for cardiovascular disease [62, 76].

Since atherosclerosis is a complex process affected by a network of numerous genes and environmental factors [77–80] and given that the effect of the C47T polymorphism in MnSOD may vary by exogenous sources of antioxidants and oxidants, multivariate regression was studied in order to identify eventual confounders such as age, gender, BMI and smoking status.

Multivariate analysis of variance revealed that Ala16 Val polymorphism and low Mn-SOD activity may be independently associated with CHD severity, a state of progressive atherosclerosis, documented by the number of vessel stenosis. Our findings point out the main role of oxidative stress in atherosclerosis.

The strong points of the present report are this is the first study to show the effect of the Ala16Val and Pro198Leu polymorphisms on CHD risk in a Tunisian population, the uniform mechanism of CHD diagnosis for all cases and the homogeneity of our population (all subjects are originated from central Tunisia). Nevertheless, the main limitation of this study is the relatively small sample size. Although there is a significant relationship between Ala16Val polymorphism and CHD; low statistical power of the current study is our another limitation. However, the finding of an association of the Ala16Val MnSOD polymorphism with CHD despite the low power means that this association is real. Indeed, with insufficient power, we may miss the true association but not to find the one that does not exist.

## Conclusion

Although small statistical power of the current study, we have demonstrated that in contrast to 198Leu variant, the 16Val variant was associated with CHD risk in men and the severity of cardiovascular events in the Tunisian population. Moreover, the SOD activity was inversely related to CHD progression as documented by the number of vessel stenosis. Further works using larger population and studying more candidate genes taking into account the gene-environment interaction remains necessary in order to better elucidate the genetic pathogenesis of CHD.

## Abbreviations

ROS: reactive oxygen species
AOX: anti-oxidants
CHD: coronary heart disease
SOD: superoxide dismutase
GPx: glutathione peroxidase
TAS: total antioxidant status
HDL: high density lipoprotein
LDL: low density lipoprotein
CAD: coronary artery disease
